# Genetic Diversity and Population Structure of F_3:6_ Nebraska Winter Wheat Genotypes Using Genotyping-By-Sequencing

**DOI:** 10.3389/fgene.2018.00076

**Published:** 2018-03-12

**Authors:** Shamseldeen Eltaher, Ahmed Sallam, Vikas Belamkar, Hamdy A. Emara, Ahmed A. Nower, Khaled F. M. Salem, Jesse Poland, Peter S. Baenziger

**Affiliations:** ^1^Department of Agronomy and Horticulture, University of Nebraska-Lincoln, Lincoln, NE, United States; ^2^Department of Plant Biotechnology, Genetic Engineering and Biotechnology Research Institute (GEBRI), University of Sadat City, Sadat, Egypt; ^3^Department of Genetics, Faculty of Agriculture, Assiut University, Assuit, Egypt; ^4^Department of Biology, College of Science and Humanitarian Studies, Shaqra University, Qwaieah, Saudi Arabia; ^5^Hard Winter Wheat Genetics Research Unit, Department of Agronomy, Kansas State University, Manhattan, KS, United States

**Keywords:** genetic variation, breeding, single nucleotide polymorphisms, population structure, *Triticum aestivum* L.

## Abstract

The availability of information on the genetic diversity and population structure in wheat (*Triticum aestivum* L.) breeding lines will help wheat breeders to better use their genetic resources and manage genetic variation in their breeding program. The recent advances in sequencing technology provide the opportunity to identify tens or hundreds of thousands of single nucleotide polymorphism (SNPs) in large genome species (e.g., wheat). These SNPs can be utilized for understanding genetic diversity and performing genome wide association studies (GWAS) for complex traits. In this study, the genetic diversity and population structure were investigated in a set of 230 genotypes (F_3:6_) derived from various crosses as a prerequisite for GWAS and genomic selection. Genotyping-by-sequencing provided 25,566 high-quality SNPs. The polymorphism information content (PIC) across chromosomes ranged from 0.09 to 0.37 with an average of 0.23. The distribution of SNPs markers on the 21 chromosomes ranged from 319 on chromosome 3D to 2,370 on chromosome 3B. The analysis of population structure revealed three subpopulations (G1, G2, and G3). Analysis of molecular variance identified 8% variance among and 92% within subpopulations. Of the three subpopulations, G2 had the highest level of genetic diversity based on three genetic diversity indices: Shannon’s information index (*I*) = 0.494, diversity index (*h*) = 0.328 and unbiased diversity index (uh) = 0.331, while G3 had lowest level of genetic diversity (*I* = 0.348, *h* = 0.226 and uh = 0.236). This high genetic diversity identified among the subpopulations can be used to develop new wheat cultivars.

## Introduction

Wheat (*Triticum aestivum* L.) is a staple cereal crop for people worldwide. Bread wheat is an allohexaploid with three distinct genomes (AABBDD) (Dubcovsky and Dvorak, 2007; Shewry, 2009; Matsuoka, 2011). The genome size of wheat is very large compared to many other species. The haploid DNA content of bread wheat genome is relatively larger than *Arabidopsis thaliana*, (∼100 times), *Oryza sativa* L. (∼40 times), and *Zea maize* L. (∼60 times) (Bennett and Smith, 1976; Arumuganathan and Earle, 1991; Sorrells, 2003).

DNA markers are extensively used in various genetic analyses including genetic diversity, evolutionary origin, genome wide association mapping, fingerprinting, and breeding applications. Single nucleotide polymorphisms (SNPs) and simple sequence repeats (SSRs) are the most common DNA marker for genetic studies.. (Rafalski, 2002). However, SNPs are excellent markers for genomic approaches particularly, for those studies that require a high number of markers such as marker-trait association, genomic selection, determining population structure, QTL mapping, and in map-based cloning (Kumar et al., 2012). Recently new techniques for genotyping using next-generation sequencing (NGS) have been developed in the large genome species as an useful method to produce high-density genome wide markers with low cost per-sample (Elshire et al., 2011). Genotyping-by-sequencing (GBS) uses the benefits of restriction enzymes to reduce the complexity of a large genome, (Poland J. et al., 2012). Poland J. et al. (2012) developed a GBS protocol that uses two restriction enzymes *(PstI/MspI)* to greatly reduce genome complexity and create more homogeneous libraries for sequencing than the one enzyme protocol (Elshire et al., 2011). The GBS method has many advantages including low cost, easy sample handling, and fewer purification steps (Davey et al., 2011).

A new cultivar release in Nebraska Wheat Breeding programs must meet minimum of these four criteria: acceptable end-use quality, stem rust (incited by *Puccinia graminus* Pers.:Pers. f. sp. *tritici* Eriks. E. Henn.) resistance, exceptional winter hardiness and improved agronomic performance relative to existing cultivars. Generally, single and three-way crosses are used in this wheat breeding program to develop new breeding populations. At least one parent of the single cross will be a Nebraska-developed or adapted line and usually two parents of the three-way cross will be from Nebraska or Kansas and selected for good end-use quality (Baenziger et al., 2001). In the Nebraska wheat breeding program, the early generations (F_2_, F_3_, and F_3:4_) are grown in one location (Lincoln or Mead NE). The F_3:5_ lines are planted in an observation nursery at Lincoln and Mead NE for selection (end-use quality, agronomic performance and disease screening). Starting from the F_3:6_, lines are tested in eight locations in Nebraska in an augmented design as the preliminary yield trial. Based on traits such as plant height, grain yield, days to flowering, grain volume weight, resistance to disease and insects, end-use quality and genomic selection for yield, 57 lines are selected and advanced to the advanced yield trial (F_3:7_ Nebraska Triplicate Nursery). In the next growing season, approximately 25 F_3:8_ lines are advanced to the elite yield trial (Nebraska Intrastate Nursery, NIN) where they continue to be tested until they are released or discarded. Nebraska breeding program uses this protocol each year to release the best line(s) which contain high levels of winter hardiness, disease resistance, agronomic performance, and good end-use quality. (Baenziger et al., 2011). The importance of our F_3:6_ lines are that they are grown in the first multilocation trial and are the gateway to all of the advanced breeding trials. Consequently, having genetic diversity in the F_3:6_ lines is critical for the breeding program, both in the lines which are advanced and for future parents selected for crossing (Baenziger et al., 2011). Genetic diversity is also important for further studies such as genome wide association study, marker-assisted selection and genomic selection.

The objectives of this study were to (i) characterize the allelic and genetic diversity, and population structure of Nebraska winter wheat genotypes at the F_3:6_ generation using genotyping by sequencing, and (ii) consider how plant breeding and selection may have affected the genetic diversity and population structure.

## Materials and Methods

### Plant Material

A set of 270 F_3:6_ lines (Nebraska Duplicate Nursery) were derived from 800 to 1000 crosses among Nebraska’s adapted cultivars or lines. The parents of these crosses are from wheat breeding programs in the Great Plains, and fewer crosses to globally important wheat lines. The breeding lines used in this study were derived from 80 crosses of the over 800 that were initially made. Pedigree information is presented in Supplementary Table 1. In fall 2016, the F_3:6_ Nebraska Duplicate Nursery were grown in nine locations (Mead, Lincoln, Clay Center, North Platte, Grant, McCook, Sidney, and Alliance in Nebraska, and one location in Kansas,). Each year was the start of a new crossing, breeding, and selection cycle. Moreover, the F_3:6_ nurseries are genotyped using GBS each year.

### DNA Extraction and Genotyping-By-Sequencing (GBS)

DNA was isolated and purified from the wheat leaves of 2–3 young 2-week-old seedlings using BioSprint 96 DNA Plant Kits (QIAGEN, Valencia, CA, United States) following the manufacturer’s instructions. The GBS method was performed based on the protocol of Poland J.A. et al. (2012). The SNPs were called using Tassel v5.2.40 GBS analysis pipeline with default parameters (Bradbury et al., 2007). The reference genome v1.0 of the ‘Chinese Spring’ genome assembly from International Wheat Genome Sequencing Consortium (IWGSC) was used in SNP calling. The SNPs were filtered according to Zhang et al. (2015), Hussain et al. (2017) based on the following criteria : (1) variant should be bi-allelic SNPs, (2) SNPs having more than 20% missing information were excluded, (3) genotypes having more than 20% missing information were excluded, and (4) the markers with minor allele frequency (MAF; MAF > 0.05) were retained. As a result, a set of 230 genotypes were remained. The raw sequence of the 230 genotypes of the current study along with 6,791 other genotypes previously genotyped in our program were combined and analyzed together in order to increase the coverage of the genome and read depth, and 206,620 SNPs were identified. As a result of these filters, 25,566 SNPs were used in this study (Supplementary Table 2).

### Data Analysis

#### Genetic Properties of Markers

The summary statistics of all 25,566 SNP markers such as gene diversity (GD), polymorphism information content (PIC), minor allele frequency (MAF), and observed heterozygosity were calculated using PowerMarker software V 3.25 (Liu and Muse, 2005). The PIC was calculated using the formula, according to (Botstein et al., 1980).

$$\begin{array}{c} \mathrm{PIC}\;=1-\;\overset{n}{\underset{j=1}{\Sigma }}P_{\mathrm{ij}}^{2}\;-\overset{n=1}{\underset{j=1}{\Sigma }}\overset{n}{\underset{k=j+1}{\Sigma }}2P_{\mathrm{ij}}^{2}P_{\mathrm{ik}}^{2} \end{array}$$

where P_ij_ and P_ik_ are the frequencies of j_th_ and k_th_ alleles for marker i, respectively.

#### Analysis of Population Structure

A model-based (Bayesian) method with 25,566 SNPs was utilized to evaluate the possible number of subpopulations in the current lines. The analysis of population structure was performed using STRUCTURE 3.4.0 (Pritchard et al., 2000). Structure was analyzed by means of *k*-values (an assumed fixed number of subpopulations) from 1 to 10 in the population. Three independent analyses were used for each *k*-value and the program was set on 100,000 as burn-in iteration, followed by 100,000 Markov chain Monte Carlo (MCMC) replications after burn-in (Chen et al., 2012) and (Zorić et al., 2012). The best *k* for the current population was determined by STRUCTURE HARVESTER (Earl and vonHoldt, 2012).

Principal coordinates analysis (PCoA) was performed based on genetic distance among the lines. The genetic distance between markers was calculated using a simple matching coefficient by R-package ‘ade4’ (Pene, 2013). Additionally, a cluster analysis of all genotypes based on genetic distance using an unweighted pair group method with arithmetic mean (UPGMA) was used.

### Analysis of Molecular Variance (AMOVA) and Genetic Diversity Indices

On each chromosome, the markers having a PIC value from 0.20 to 0.37 were selected and a total of 7,842 SNPs were used for AMOVA. The number of subpopulations determined based on STRUCTURE results were used for AMOVA.

The genetic indices such number of loci with private allele, number of different alleles (Na), number of effective alleles (Ne), diversity index (*h*), unbiased diversity (uh), and Shannon’s Information Index (*I*) were calculated. The AMOVA and estimation of genetic indices were performed using GeneAlEx 6.41 (Peakall and Smouse, 2006).

## Results

### Distribution of SNPs on the Different Genomes of Wheat

**FIGURE 1:**
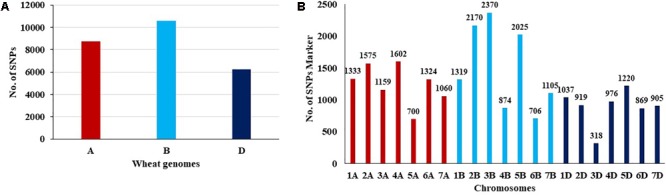
**(A,B)** Distribution of SNP makers across all chromosomes in the wheat genomes A,B, and D.

The number of SNPs identified was, 8,753, 10,569, and 6,245 in genomes A, B, and D, respectively (**Table 1** and **Figure 1A**). The number of SNPs per chromosome ranged from 319 (chromosome 3D) to 2,370 (chromosome 3B). The lowest and highest number of SNPs identified per chromosome in each genome ranged from 700 (Chr. 5A) to 1575 (Chr. 2A), 706 (Chr. 6B) to 2,370 (Chr. 3B), and 319 (Chr.3D) to 1,220 (Chr. 5D) (**Figure 1B**).

**Table 1:** Distribution of SNP markers by chromosome across hexaploid wheat genomes.

	Genome			
Chromosome No.	A	B	D	Total
1	1333	1319	1037	3689
2	1575	2170	919	4664
3	1159	2370	319	3848
4	1602	874	976	3452
5	700	2025	1220	3945
6	1324	706	869	2899
7	1060	1105	905	3070
**Total**	**8753**	**10569**	**6245**	**25567**

### Genetic Diversity and PIC

**FIGURE 2:**
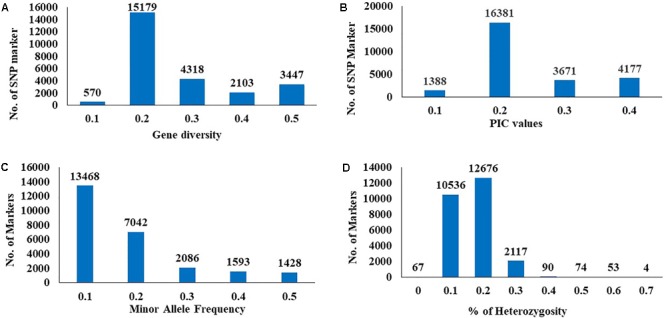
Distribution of genetic diversity **(A)**, polymorphic information content (PIC) **(B)**, minor allele frequency **(C)**, and percentage of heterozygosity **(D)** for 25567 SNP marker in the 230 Nebraska wheat genotypes.

The GD and PIC value across all chromosomes varied from 0.1 (570 SNPs) to 0.5 (3,447 SNPs) with an average 0.3 and from 0.1 (1,338 SNPs) to 0.4 (4,177 SNPs) with an average of 0.25, respectively (**Figures 2A,B**). A similar range of GD and PIC values were observed in the three wheat genomes. Most markers (13,468 SNPs) had a minor allele frequency of 0.1 (**Figure 2C**). The observed heterozygosity ranged from 0.1 (10,536 SNPs) to 0.7 (4 SNPs) (**Figure 2D**).

### Population Structure and Relationships

**FIGURE 3:**
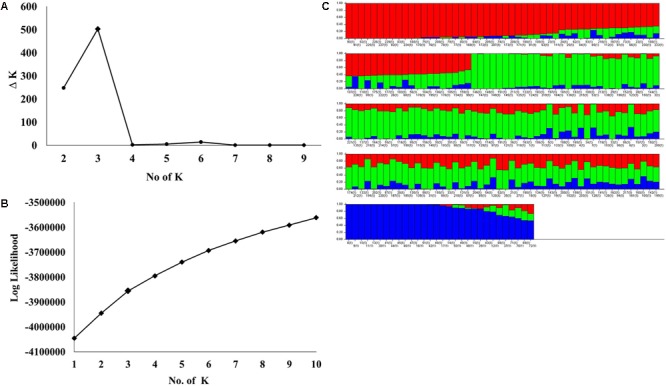
**(A)** Delta (Δ)K for differing numbers of subpopulations (*k*), **(B)** the average of log-likelihood value and **(C)** estimated population structure of 230 wheat genotypes on (*k* = 3), G refers to subpopulation using STRUCTURE.

**FIGURE 4:**
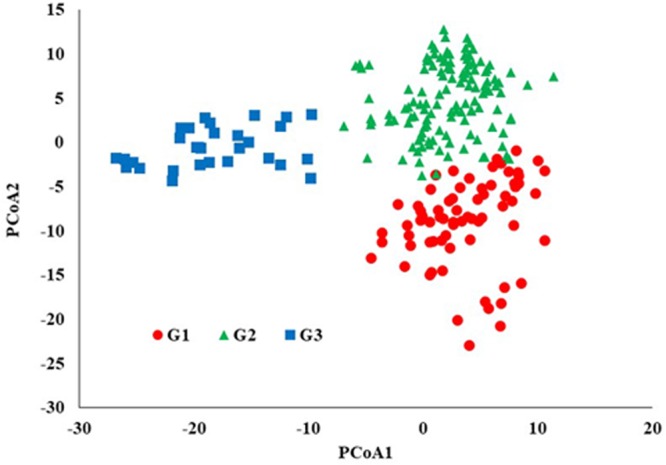
Principal coordinates analysis (PCoA) based on genetic distance (SNPs).

STRUCTURE analysis software was used to study the population structure of 230 genotypes from Nebraska Duplicate Nursery (**Figure 3**). In order to find the suitable value of *K*, the number of clusters (*K*) was plotted against *ΔK* which showed a sharp peak at *k* = 3 (**Figure 3A**). Remarkably, a continuous-gradual increase was observed in the assessed log likelihood [LnP(D)] with the increase of *K* (**Figure 3B**) and the best number of *K* which clearly defined the number of populations was *K* = 3, indicating that three subpopulations could include all the 230 of Nebraska winter wheat genotypes with the highest probability. Three subgroups (**Figure 3C**) were identified and comprised 70, 130, and 30 genotypes in G1, G2, and G3, respectively (**Table 2**). The results of PCoA agreed with STRUCTURE analyses by clustering the 230 winter wheats genotypes into three clear groups (**Figure 4**).

**Table 2:** The results of STRUCTURE analysis of 230 lines in the Nebraska breeding program for the fixation index (Fst) (significant divergences), average distances (expected heterozygosity) and number of genotypes in each subpopulation.

Population	Fst^1^	Exp. hetero^2^	No. of Genotypes
G1	0.1539	0.1979	70
G2	0.1078	0.2176	130
G3	0.2437	0.1746	30

*^*1*^Fst is a measure of genetic differentiation; ^*2*^Expected heterozygosity.*

A significant genetic divergence was observed among subpopulations and average distance (expected heterozygosity) among genotypes in each subpopulation (**Table 2**). The highest value of expected heterozygosity was found in G2 with a value of 0.217 and the lowest value was found in G3 with a value of 0.174 while the G1 had a moderate expected heterozygosity with an average of 0.197. The Fixation index (Fst) was calculated to test the population substructure and it is considered the most efficient index for assessing the overall genetic variation among subpopulations. So, the Fst value were 0.107, 0,153, and 0.234 for G2, G3, and G1.

### Genetic Differentiation of Populations

The three subpopulations identified in STRUCTURE analysis were used in GenAlex 6.41 software to calculate the AMOVA and the genetic diversity indices. The AMOVA, Fst and Nm or haploid number of migrants (Nm) are provided in **Table 3**. The AMOVA revealed that 8% of the total variation was found among subpopulations, while, the rest of variation (92%) was within subpopulations. The haploid Nm was very high (5.621) indicating a high gene exchange among subpopulations. These results demonstrated that genetic differentiation among subpopulations was low and within subpopulations was high. The UPGMA based on genetic distance among all genotypes was performed again with the 7,842 SNPs (Supplementary Figure 2). The results revealed three subgroups which are in an agreement with the population structure analysis (with 25, 566 SNPs).

**Table 3:** Analysis of molecular variance using 25, 566 SNPs of the genetic differentiation among and within three subpopulations of 230 F_3:6_ lines in the Nebraska breeding program.

Source	df	SS	MS	Est. Var.	%	*P* value
Among Pops	2	27537.972	13768.986	177.452	8%	0.001
Within Pops	227	480979.071	2118.851	2118.851	92%	0.001
Total	229	508517.043		2296.303	100%	0.001

Fixation index (Fst)	0.082					
Nm (haploid)	5.621					

### Allelic Pattern Across the Populations

**FIGURE 5:**
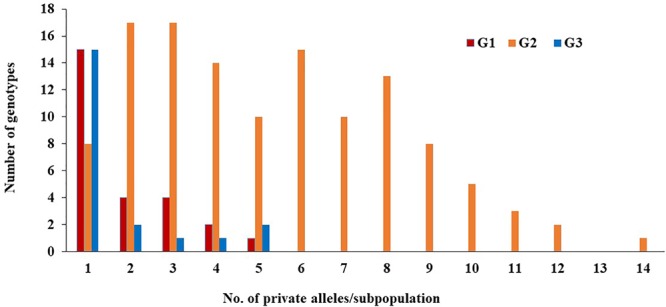
Number of private alleles in each subpopulation (G).

Of the 230 genotypes, 170 (74%) were found to possess several loci with private alleles (**Figure 5**). The number of genotypes with private alleles were 26 (37%), 123 (95%), and 21 (70%) in G1, G2, and G3, respectively. The number of loci with private alleles ranged from 1 to 5 in G1 and G3 subpopulations, while, it ranged from 1 to 14 in G2 subpopulation.

The mean value of different alleles (Na) and Number of effective alleles of the three subpopulations (**Table 4**) were 1.898 and 1.480, respectively. Populations had average values of 0.433, 0.286, and 0.292 for *I, h*, and uh, respectively (**Table 4**). Of the three subpopulations, G2 population was the most diverse (*I* = 0.494, *h* = 0.328 and uh = 0.331), whereas the sub-population G3 was the least diverse (*I* = 0.348, *h* = 0.226, and uh = 0.236). The percentage of polymorphic loci per population ranged from 91.85% (G3) to 98.36% (G2) with an average of 95.69%.

**Table 4:** Mean of different genetic parameters including number of different alleles (Na), number of effective allele (Ne), Shannon’s index (*I*), diversity index (*h*), unbiased diversity index (uh), and percentage of polymorphic loci (PPL) in each subpopulation of the 230 lines.

Subpopulations	Na	Ne	*I*	*h*	uh	PPL
G1	1.962	1.510	0.459	0.302	0.307	96.85%
G2	1.982	1.555	0.494	0.328	0.331	98.36%
G3	1.751	1.375	0.348	0.226	0.236	91.85%
Mean	1.898	1.480	0.433	0.286	0.292	95.69%

*Shannon’s information index = -1^∗^Sum[Pi^∗^Ln(Pi)], h. Diversity = 1- Sum Piˆ2, uh Unbiased diversity = [N/(N–1)]^∗^h. Where Pi is the frequency of ith alleles for the population and Sum Piˆ2is the sum of squared population allele frequencies and PPL = Σ Pi/N, 1 = 1 where Pi = proportion of loci polymorphic in a population and N = number of populations.*

## Discussion

The 25,566 SNPs markers were identified across the three wheat genomes A, B, and D (**Figure 1**). The highest number of markers were found in B genome, while, the lowest were in the D genome indicating lower diversity in the D genome. Pour et al. (2017) genotyped a set of 369 Iranian hexaploid wheat accessions using 16,506 GBS-SNP and found that the highest number of SNPs were on B genome, while, D genome had the lowest number of SNPs. The D genome had the fewest polymorphic markers, indicating a lower genetic diversity due to the low frequency of recombination rates (Chao et al., 2009). The diversity proportion in *T. aestivum* came from the polyploid nature of its tetraploid ancestor. This result can be interpreted as at the nucleotide level of A and B genomes, the average diversity was estimated to be 30% of that existed in wild emmer. Hexaploid wheat was found to possess a larger portion of the natural gene diversity found in its tetraploid ancestor (AABB) than of the diversity found in *Aegilops tauschii* (DD) (Dubcovsky and Dvorak, 2007). The contribution of A, B, and D genomes to genetic variation was also discussed in earlier studies using different types of markers such as, DArT (Nielsen et al., 2014), SSRs (Röder et al., 1998), RFLPs (Liu and Tsunewaki, 1991), and AFLP (Peng et al., 2000) in common hexaploid wheat as well as in *T. dicoccoides* (Li et al., 2000; Thuillet et al., 2005). Our results confirm that GBS derived SNP can be used for genetic studies in wheat breeding programs.

Genetic diversity and PIC values are extremely helpful for studying the level of polymorphisms among genotypes in plant breeding programs. In this study, the average of GD and PIC were 0.3 and 0.25, respectively, (**Figure 2**). Botstein et al. (1980) reported that the PIC values can be classified in to three categories (1) if the PIC value of the marker is more than 0.5, the marker is considered a highly informative, (2) if the PIC value ranged from 0.25 to 0.5, the marker is a moderately informative, and (3) if the PIC value less than 0.25, then the marker is slightly informative marker. The PIC value of SNP marker is considered moderate or low informative markers. The primary reason for this result is due to the bi-allelic nature of the SNPs which is restricted to extreme PIC values of 0.5 (when the two alleles have identical frequencies). The PIC value in multi-allelic markers (e.g., SSR), usually is more than 0.5. The second reason is due to nucleotide mutation rate in intronic regions of the wheat genome (4–8 × 10^-9^) substitutions nt^-1^ year^1^ (Dvorak and Akhunov, 2005). This mutation rate in GBS-SNPs is slower than SSRs mutation rate (2.4 × 10^-4^) (Thuillet et al., 2002). The low mutation rate in SNPs could lead to the low correlation observed between the genetic distances. The PIC value obtained in this study were in close agreement to the mean PIC value of 0.27 estimated previously (Somers et al., 2003; Chao et al., 2009; Edwards et al., 2009; Allen et al., 2011). The distribution of informative markers is important to reflect the overall diversity in the crop populations (Nielsen et al., 2014). The PIC values are a good indication of informative markers which can be used for genotyping plant populations and studying genetic diversity (Salem and Sallam, 2016).

### Population Structure and Relationships

Population structure analysis is useful in understanding genetic diversity and association mapping studies. The Nebraska wheat lines were divided to three subgroups. The PCoA results were in a agreement with STRUCTURE results. Moreover, the dendrogram analysis (UPGMA) gave the same results (Supplementary Figure 1). The presence of structure in the current population was not surprising for two reasons. First, due to the pedigree of the genotypes (Supplementary Table 1), the genotypes in each subpopulation were largely derived from common parents. The G2 subpopulation had the highest number of genotypes, followed by G1, and then G3. The number of common parents in G1 and G3 was higher than those common in G2. Of course, there were some common parents between G1 and G2. Subpopulation G3 shared very few common parents with G2. These results suggested the higher number of genotypes in G1 and G2 compared to G3 was due to similar parental genotypes. The second reason is most likely due to breeder selection based on specific traits (e.g., yield, stem rust resistance, herbicide tolerance, etc) according to Nebraska wheat breeding program objectives. El-Basyoni et al. (2013) studied the genetic diversity in the *F*_3:6_ Nebraska Duplicate Nursery in 2010 and 2011. They also found three subpopulations. As the 2010 and 2011 nurseries were separated by at least 6 years from the 2017 nursery and numerous crosses (approximately 1,000 crosses per year) were made between the nurseries, it was interesting to again find three subpopulations in each nursery from different years and different pedigrees. Presumably the role of breeder selection and the agroecological zones of Nebraska fostered the formation of the three subpopulations and the maintenance of this genetic diversity. Maintaining genetic diversity is critical for continued breeding advances.

Selection and genetic drift can cause the presence of subgroups in the large populations (Buckler and Thornsberry, 2002; Breseghello and Sorrells, 2006). Studying population structure is important for genome wide association studies (GWAS) which are used to identify marker-traits associations for important traits. The presence of structure in the mapping population could cause spurious association between traits and marker alleles (Oraguzie et al., 2007). Therefore, testing the population structure is the first step before conducting GWAS to identify a true association between a trait of interest and makers alleles which could lead to identification of underlying genes.

### Genetic Differentiation of Populations

The three subpopulations presented a remarkable differentiation in the loci having private alleles. The G2 subpopulation had a higher number and a wide range of loci with private alleles than the other two subpopulations (G1 and G3). This result is probably due to G2 being the largest group and also most genetically diverse compared with the other two subgroups G1 and G3. The advantage of calculating private alleles was that it provided information on alleles that existed in only one subpopulation. G2 had 14 private alleles which was more than those found in G1 and G3 indicating the high genetic diversity that existed among the genotypes of G2. The private allele data provide useful information on unique genetic variability in certain loci and identifying highly -diverse genotypes that could be used in crop breeding programs as parental lines in order to maximize the allele richness in the population (de Oliveira Borba et al., 2009; Salem and Sallam, 2016). Another reason of the higher genetic diversity among genotypes in G2, may be due to the origin of the common parents of the G2 genotypes which represented many different breeding programs in United States (such as the breeding programs in Colorado, Oklahoma, South Dakota, Missouri, Kansas, and Nebraska), CIMMYT, China, South Africa and Turkey. The common parents of the genotypes in G1 and G3 were mainly from South Dakota and Nebraska. Remarkably, all genotypes in G3 shared one common parent (NW03666), while, in G1, three common parents NW07534, NE06545 (Baenziger et al., 2014) and NE05426 were shared. As a breeder, the subpopulations reflected a group which represented our main breeding pool (G2) for southern Nebraska and the southern Great Plains, a group that is oriented to white wheat (G3), and a group that is most likely adapted to northern Nebraska and states further north (G1). The pedigree information and breeder selection target/objective offered some clarification or explanation of the biological meaning for the presence of these three subgroups (Supplementary Table 1).

The results of AMOVA indicated high level of genetic diversity within-subpopulations. Although the variation among subpopulations was low (8%), it was significant according to the partitioning value (*P* < 0.001). The selection for specific agronomic traits in the breeding program of the Nebraska winter wheat was considered the main reason for this high variation within groups. The low level of diversity among subpopulations was due to the high value of gene flow. (Arora et al., 2014). Wright (1965) reported that the Nm (haploid) value less than one indicates limited gene exchange among subpopulations. In the current study, the Nm value (5.621) was very high, therefore the genetic exchange among subpopulations led to a low genetic differentiation among subpopulations as would be expected within a breeding program.

Selection and gene flow were the main causes of population structure in the current population. The high gene flow was due to the common parents which are from different regions (especially the common parents of G2). These main, hence common, parents were involved in many crosses each year in Nebraska breeding program. The progenies were elite lines and these parents were exposed to selection based on four criteria as described previously.

The allelic pattern and genetic diversity indices provided useful information on genetic diversity in each subpopulation. Although the three subpopulations had observable diversity, G2 had the highest genetic diversity. The understanding of genetic diversity within the Nebraska winter wheat breeding program will be helpful for planning future studies and maintaining and monitoring genetic diversity in a breeding program.

## Conclusion

In this study, we employed high-throughput SNP genotyping to explore the utility of SNP markers for genomic analyses in elite wheat breeding lines. Although the elite genotypes were highly selected, they were genetically diverse. This level of genetic diversity can be used for future breeding progress to devlop new wheat cultivars with desirable characteristics such as high yield potential, tolerance to biotic and abiotic stress, and good end-use quality while being adapted to diverse environments. Moreover, our study identified three subpopulations which could be explained by their parentage and selection history. The G2 subpopulation has largest number of genotypes, exhibited the highest values for private alleles, *I, h*, uh parentage of polymeric loci, and was the most diverse. Using this knowledge of population structure and genetic diversity are very important for future genetic analyses such as genomic selection studies, GWAS and marker-assisted selection which support the main objectives in Nebraska breeding program to genetically improve winter wheat.

## Author Contributions

SE conducted the experiments of this study, performed all the statistical analysis, and wrote the manuscript. AS designed the study, helped in the statistical analyses, and drafted the manuscript. VB performed the SNP calling and discussed the results. HE, AN, and KS helped in discussing the results and drafting the paper. JP performed the GBS for the population. PB designed the study, discussed the results, and helped draft the manuscript.

## Conflict of Interest Statement

The authors declare that the research was conducted in the absence of any commercial or financial relationships that could be construed as a potential conflict of interest.
