# Supplementary figures and images for: Genetic Diversity and Population Structure of F3:6 Nebraska Winter Wheat Genotypes Using Genotyping-By-Sequencing
Source: Front Genet. 2018 Mar 12;9:76. doi: 10.3389/fgene.2018.00076 (PMC5857551; doi:10.3389/fgene.2018.00076)

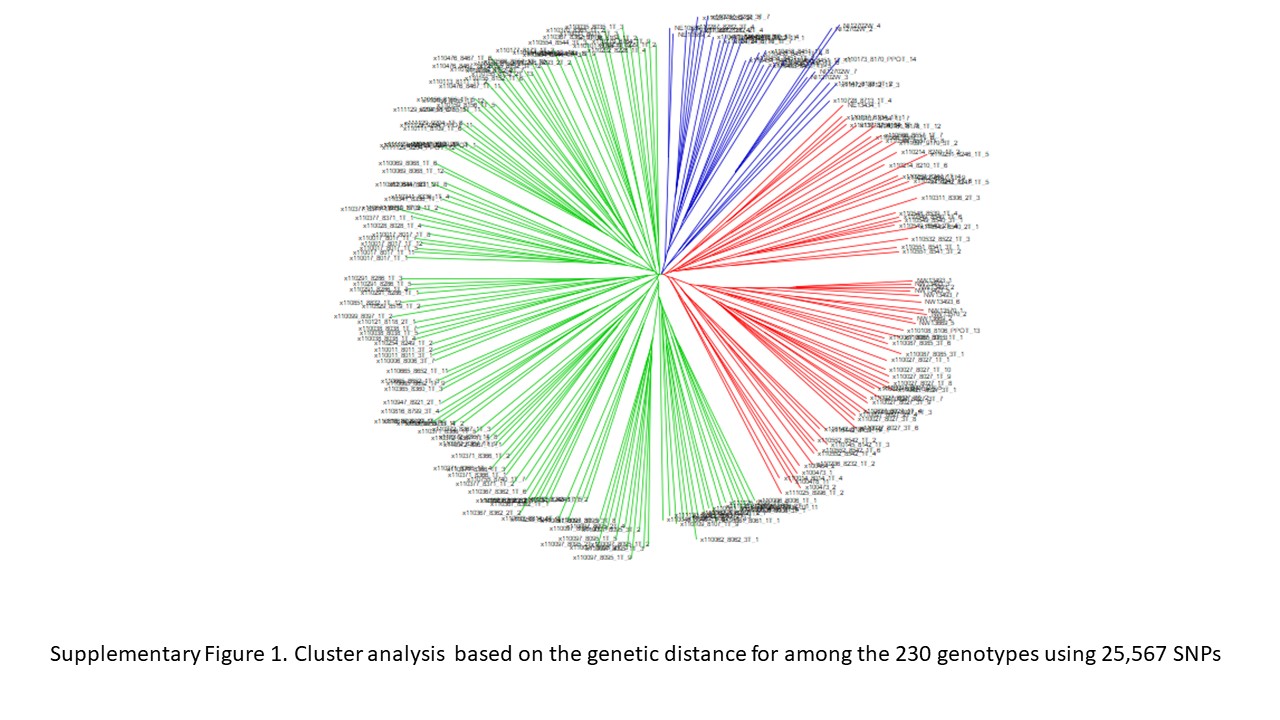

Supplement: Supplementary file 2 [file Image_1.JPEG]

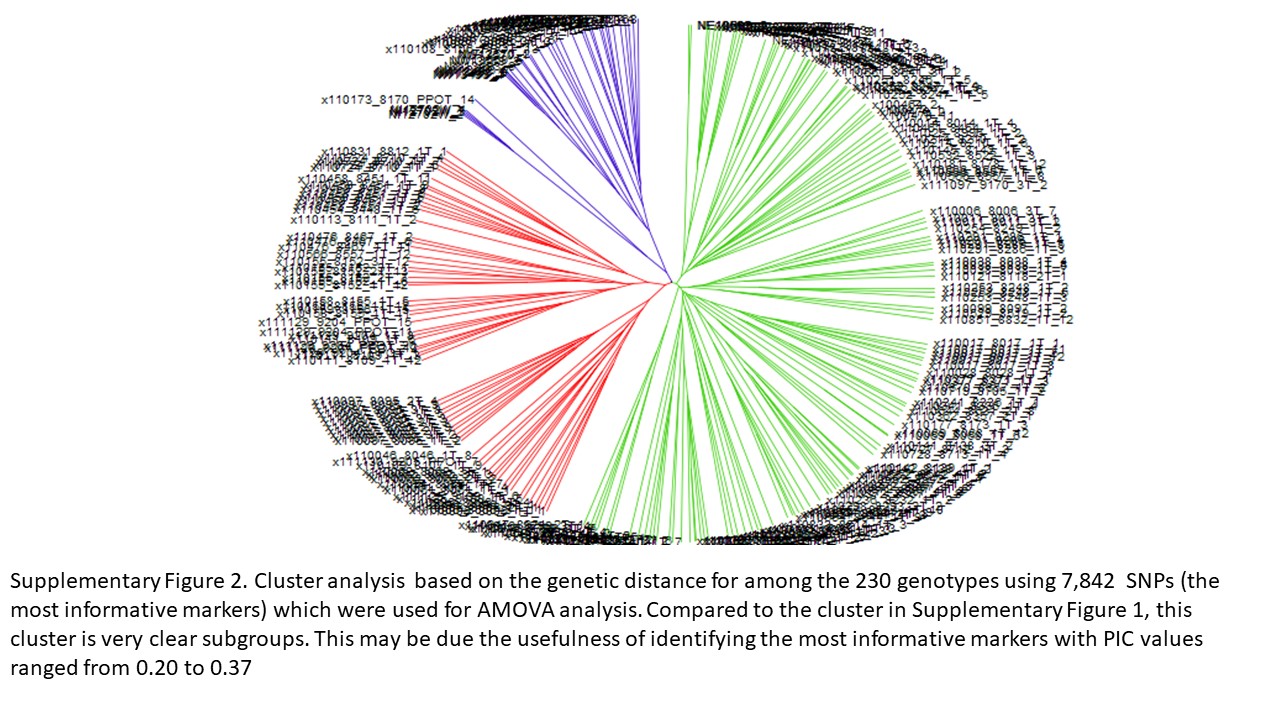

Supplement: Supplementary file 3 [file Image_2.JPEG]
